# Distinct patterns of association between the hemoglobin glycation index, the stress–hyperglycemia ratio, and the risk of new-onset atrial fibrillation in critically ill patients

**DOI:** 10.3389/fendo.2025.1656783

**Published:** 2025-09-17

**Authors:** Qiqiang Jie, Gang Li, Weichun Qian, Mingzhu He, Haibo Jia, Fengfu Zhang, Jianping Wang

**Affiliations:** ^1^ Department of Cardiology, Nanjing First Hospital, Nanjing Medical University, Nanjing, China; ^2^ Department of Cardiology, The First Affiliated Hospital of Zhengzhou University, Zhengzhou, China; ^3^ Department of Geriatrics, Nanjing First Hospital, Nanjing Medical University, Nanjing, China

**Keywords:** hemoglobin glycation index (HGI), stress–hyperglycemia ratio (SHR), new-onsetatrial fibrillation (NOAF), critically ill patients, glycemic variability

## Abstract

**Background:**

This study investigated the associations between two novel glycemic indices, the hemoglobin glycation index (HGI) and the stress hyperglycemia ratio (SHR), and the risk of new-onset atrial fibrillation (NOAF) in critically ill patients.

**Methods:**

We retrospectively analyzed data from 3,882 adults in the MIMIC-IV database, with the primary outcome defined as NOAF within 7 days of intensive care unit (ICU) admission. Multivariate Cox regression and restricted cubic spline analyses were used to evaluate associations.

**Results:**

NOAF occurred in 750 patients (19.3%). After adjustment for confounders, HGI exhibited a significant inverted U-shaped association with NOAF risk, with the highest risk in intermediate quartiles. In contrast, the SHR demonstrated a significant linear inverse relationship with NOAF risk, with higher SHR quartiles consistently associated with lower risk. These associations were especially pronounced in nondiabetic patients and remained consistent across key clinical subgroups.

**Conclusion:**

Our findings indicate that the HGI and SHR independently predict NOAF in critically ill patients and may provide valuable tools for risk stratification and personalized glycemic management in the ICU.

## Introduction

Atrial fibrillation (AF) is recognized as the most prevalent form of cardiac arrhythmia in the context of critical illness (1). A particular consequence is the development of new-onset atrial fibrillation (NOAF), a clinical phenomenon whose incidence has been documented in the literature to range from 5% to 46% in specific patient cohorts, most notably those with sepsis (2–4). The onset of NOAF often leads to severe adverse outcomes, including hemodynamic instability, an increased risk of systemic embolism and stroke, prolonged stays in the intensive care unit (ICU), and increased mortality rates (5–9).

The pathophysiology of NOAF in critically ill patients is complex and multifactorial. It involves a combination of factors, such as systemic inflammation, sympathetic nervous system overactivity, the use of vasopressors, and organ dysfunction (1, 10–12). Among these factors, stress hyperglycemia—a common metabolic response to critical illness—is increasingly recognized as a key contributor to the development of NOAF (13–18). This presents a clinical challenge: while tight glycemic control is important for mitigating adverse outcomes, it must be carefully balanced against the risk of iatrogenic hypoglycemia (19). Therefore, effective glucose management is crucial for reducing the risk of NOAF in this vulnerable population.

Traditional glycemic monitoring, which relies on single-point glucose measurements or HbA1c, often fails to capture the dynamic nature of stress-induced hyperglycemia (20, 21). To overcome this, novel indices have been developed. The hemoglobin glycation index (HGI) measures relative hyperglycemia by quantifying the difference between a patient’s actual blood glucose and their expected level on the basis of chronic glycemic status (HbA1c) (22, 23). Similarly, the stress hyperglycemia ratio (SHR) is used to assess the severity of stress hyperglycemia by calculating the ratio of admission blood glucose to HbA1c-derived average glucose (20, 21). These indices provide a more nuanced assessment of glycemic dysregulation than do absolute glucose values alone.

While emerging evidence supports the use of HGI and SHR as prognostic markers in cardiovascular diseases such as myocardial infarction and heart failure (21, 22, 24, 25), their role in the broader context of critical care is less defined. Specifically, the relationships between these novel glycemic indices and the risk of NOAF in a general population of critically ill patients have not been adequately investigated.

Therefore, using the MIMIC-IV ICU database, we finalized a statistical analysis plan before outcome modeling to (i) estimate the covariate-adjusted associations of HGI and SHR with incident NOAF; (ii) characterize dose–response shapes via prespecified restricted cubic splines; and (iii) evaluate robustness across predefined subgroups and diabetes-stratified sensitivity analyses. Guided by biological plausibility and indirect evidence from stress–glycemia metrics and cardiovascular outcomes, we prespecified that HGI may exhibit a nonlinear (inverted-U) association with NOAF, whereas SHR would display an approximately inverse association; these *a priori* hypotheses were formally tested.

## Materials and methods

### Study design and data source

This was a retrospective cohort study using the MIMIC-IV v2.2 database (ICU admissions at Beth Israel Deaconess Medical Center, 2008–2019). MIMIC contains de-identified health records maintained by the Laboratory for Computational Physiology at MIT. Institutional review board approvals were in place at MIT and BIDMC, and informed consent was waived owing to de-identification. One author completed the required training and performed all data extractions. The reporting follows the STROBE guidelines.

### Study population

**Figure 1:**
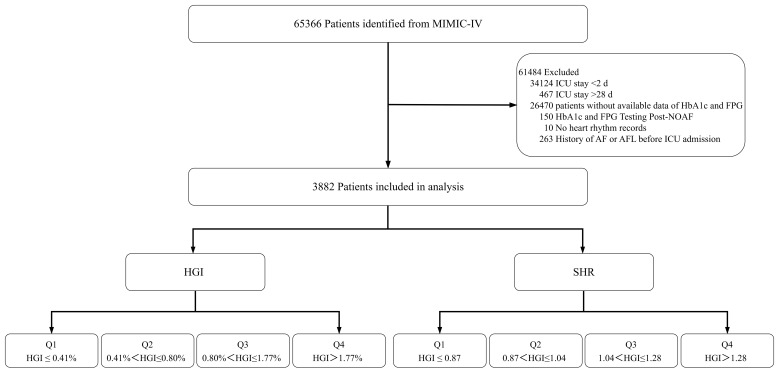
Flowchart illustrating patient selection from the MIMIC-IV database, including exclusion criteria and final stratification by quartiles of HGI and SHR. MIMIC-IV, Medical Information Mart for Intensive Care-IV; HbA1c, hemoglobin A1c; FPG, fasting plasma glucose; NOAF, new-onset atrial fibrillation; AF, atrial fibrillation; AFL, atrial flutter; ICU, intensive care unit; HGI, hemoglobin glycation index; SHR, stress hyperglycemia ratio.

We initially identified 65,366 unique patients at their first ICU admission (≥18 years). Eligibility required availability of HbA1c and an admission glucose (earliest within 12h of ICU entry), with both recorded before any AF ascertainment. We then excluded patients with (1) an ICU length of stay <2 days (n = 34,142) or >28 days (n = 467); (2) a documented history of atrial fibrillation (AF) or atrial flutter (AFL) prior to ICU admission (n = 263); (3) missing either admission glucose or HbA1c (n = 26,470); (4) missing admission glucose or HbA1c data obtained after NOAF onset (n = 150); (5) absence of any heart rhythm records during the ICU stay (n = 10); and (6) missing key demographics (age, sex, or race). After these exclusions, 3,882 patients remained for analysis. A detailed selection flowchart is shown in **Figure 1**.

### Data extraction and definitions

Data were extracted via Structured Query Language (SQL) with PostgreSQL (version 16.0). Unless otherwise specified, all baseline variables were captured within the first 24 hours of ICU admission and prior to any ascertainment of NOAF.

The primary exposure variables used were the hemoglobin glycation index (HGI) and the stress/hyperglycemia ratio (SHR). Admission glucose was defined as the first plasma glucose measurement within 12 hours of ICU admission. The HGI was calculated as follows: observed HbA1c − predicted HbA1c, where predicted HbA1c = (0.009 × admission glucose [mg/dL]) + 4.940. The SHR was calculated via consistent units as admission glucose [mg/dL]/([28.7 × HbA1c %] − 46.7). Extreme or biologically implausible SHR values (<0.1 or >15) were reviewed and excluded.

Outcome Variable: The primary outcome was incident NOAF within 7 days of ICU admission. The event time was defined as the time of the first documented episode of AF, ascertained from bedside rhythm charting or telemetry records. Patients who did not develop NOAF were censored at the time of ICU discharge, in-hospital death, or on day 7, whichever occurred first.

Covariates: We collected baseline data on covariates that were included in the fully adjusted model (Model 3): (1) demographics (age, sex, race, BMI); (2) major comorbidities (myocardial infarction, congestive heart failure, diabetes, renal disease, liver disease, prior cardiac surgery); (3) illness severity scores (SOFA, SAPS II) and acute conditions (acute kidney injury [AKI], delirium); (4) vital signs (heart rate, mean arterial pressure [MAP], respiratory rate, SpO_2_); (5) key laboratory values (white blood cell count, red blood cell count, platelet count, electrolytes, creatinine); and (6) ICU interventions (mechanical ventilation, vasopressor use, CRRT). Continuous variables were assessed for distribution and log-transformed when appropriate.

### Disease severity and comorbidity scoring

Time window and temporal ordering. All severity scores were computed from the worst recorded values within the first 24 hours after ICU admission and prior to any ascertainment of new-onset atrial fibrillation (NOAF) to minimize immortal-time and reverse-causation bias.

The Sequential Organ Failure Assessment (SOFA) score was derived across six organ systems—respiratory (PaO_2_/FiO_2_ with ventilatory support considered), coagulation (platelet count), liver (total bilirubin), cardiovascular (mean arterial pressure and vasopressor dose thresholds per the original SOFA definition), central nervous system (Glasgow Coma Scale, GCS), and renal (serum creatinine or urine output) systems. When preexisting organ dysfunction was unknown, the baseline value was assumed to be zero. For the CNS domain, we preferentially used presedation GCS when sedation information allowed a reliable estimate; otherwise, we used the recorded GCS without sedation adjustment and evaluated the impact in sensitivity analyses (see **Supplementary Methods**). Vasopressor categories followed the original SOFA thresholds (dopamine, dobutamine, epinephrine, and norepinephrine) and used weight-normalized doses.

The simplified acute physiology score II (SAPS II) was computed from 17 variables captured in the first 24 hours (the worst value used for each physiological variable): 12 physiologic measurements (temperature, heart rate, systolic blood pressure, PaO_2_ or alveolar–arterial oxygen gradient depending on FiO_2_, 24-hour urine output, serum bicarbonate, total bilirubin, serum sodium, serum potassium, blood urea nitrogen, white blood cell count, and GCS), age, admission type (medical/scheduled surgical/unscheduled surgical), and three chronic conditions (AIDS, metastatic cancer, hematologic malignancy). Per the original specification, the oxygenation component used PaO_2_ when FiO_2_ < 0.5 and the A–a gradient when FiO_2_ ≥ 0.5; we followed this rule in our implementation. As with the SOFA, we used presedation GCSs where feasible; otherwise, the recorded value was used, and robustness was assessed via sensitivity analyses.

The comorbidity burden was quantified via the Charlson Comorbidity Index mapped from ICD-9/ICD-10 diagnosis codes recorded during the index hospitalization via the Quan et al. coding algorithm; weights were summed to obtain the CCI. Because using only index-stay diagnoses can underascertain preexisting conditions, we flag this as a limitation and probed robustness in sensitivity analyses (e.g., restricting diagnoses flagged as present-on-admission [POA], where available).

### Missing data management

Missing covariate data were handled via multiple imputation by chained equations (MICE; m=5; 10 iterations) under a missing−at−random assumption, including all variables in the fully adjusted model (Model 3). The exposure and outcome variables were not imputed. The estimates were pooled via Rubin’s rules.

### Statistical analysis

For descriptive analyses, baseline characteristics were compared between patients with and without NOAF and across the HGI and SHR quartiles. Continuous variables, presented as medians with interquartile ranges (IQRs), were compared via the Mann–Whitney U test for two-group comparisons and the Kruskal–Wallis test for comparisons across quartiles. Categorical variables are presented as counts and percentages and were compared via the chi-square test or Fisher’s exact test, as appropriate.

The cumulative incidence of NOAF across quartiles was visualized with Kaplan–Meier curves and compared via the log-rank test. We then used Cox proportional hazards models to estimate hazard ratios (HRs) and 95% confidence intervals (CIs) for the associations between HGI/SHR quartiles and NOAF risk. A prespecified, progressive adjustment strategy was used: Model 1: Unadjusted. Model 2: Adjusted for age, sex, race, and BMI. Model 3 (Fully Adjusted): Further adjusted for SOFA and SAPS II scores, major comorbidities, key ICU interventions, vital signs, and laboratory parameters, as listed in the definitions section.

To assess nonlinear relationships, we modeled HGI and SHR as continuous variables via restricted cubic splines with three knots (at the 10th, 50th, and 90th percentiles). A likelihood ratio test was used to calculate a P value for nonlinearity. The proportional hazards assumption was checked via Schoenfeld residuals, and multicollinearity was assessed via variance inflation factors (VIFs). All the statistical analyses were prespecified and conducted via R software (version 4.4.0). A two-sided P value < 0.05 was considered statistically significant.

### Subgroup and sensitivity analyses

Prespecified subgroup analyses assessed the consistency of associations across age (≥65 vs <65 years), sex, BMI (≥30 vs <30 kg/m²), race, and history of myocardial infarction, congestive heart failure, diabetes, and prior cardiac surgery. Subgroup estimates were obtained from the fully adjusted Model 3, using the same exposure parameterizations (quartiles and, where applicable, continuous RCS). Effect modification was evaluated on the multiplicative scale by adding exposure × subgroup cross-product terms to Model 3; P for interaction was derived from likelihood-ratio tests comparing models with and without the interaction. The results are displayed with forest plots. The primary sensitivity analysis prespecified stratification by diabetes status, repeating the full modeling framework within each stratum.

## Results

### Baseline characteristics of the study population

A total of 3882 patients were enrolled in the analysis. Baseline characteristics are shown in **Table 1**. The median age was 68 years, and 2255 (58.1%) patients were male. Compared with those without NOAF, patients with NOAF were older and had a greater comorbidity burden (higher Charlson Comorbidity Index scores and a greater prevalence of congestive heart failure, myocardial infarction, and renal disease (all p < 0.01)). Greater severity (higher SAPS II and SOFA scores) led to higher requirements for intensive interventions (vasopressor use, mechanical ventilation, and continuous renal replacement therapy) in patients with NOAF (all p < 0.001). Vital signs were more unstable in patients with NOAF (significantly lower blood pressure). Key laboratory data revealed lower red blood cell counts, lower platelet counts, and metabolic alterations (lower glucose, calcium, and SHR levels (all p < 0.01)) (**Table 1**, **Supplementary Table S1**).

**Table 1:** Baseline characteristics of critically ill patients with and without new-onset atrial fibrillation (NOAF): core variables used in multivariable models.

Characteristic		NOAF		P value
	Overall N = 3,882	No N = 3,132	Yes N = 750	
Demographics				
Age, y	68 (57, 77)	66 (55, 76)	75 (66, 82)	<0.001
Gender, No. (%)				0.641
Female	1,627 (41.9%)	1,307 (41.7%)	320 (42.7%)	
Male	2,255 (58.1%)	1,825 (58.3%)	430 (57.3%)	
Race, No. (%)				<0.001
Black	377 (9.7%)	336 (10.7%)	41 (5.5%)	
White	2,144 (55.2%)	1,675 (53.5%)	469 (62.5%)	
Other	1,361 (35.1%)	1,121 (35.8%)	240 (32.0%)	
BMI, kg/m²	28.9 (26.2, 31.2)	28.9 (26.5, 31.0)	28.5 (25.1, 32.0)	0.102
Disease Severity Scores				
SOFA, score	1.00 (0.00, 2.00)	1.00 (0.00, 2.00)	1.00 (0.00, 4.00)	<0.001
SAPS_II, score	33 (25, 41)	31 (24, 40)	37 (31, 46)	<0.001
CCI, score	5.00 (3.00, 7.00)	5.00 (3.00, 7.00)	6.00 (4.00, 7.00)	<0.001
Therapeutic Interventions				
CRRT, No. (%)				<0.001
No	3,734 (96.2%)	3,039 (97.0%)	695 (92.7%)	
Yes	148 (3.8%)	93 (3.0%)	55 (7.3%)	
Vasopressor, No. (%)				<0.001
No	2,512 (64.7%)	2,188 (69.9%)	324 (43.2%)	
Yes	1,370 (35.3%)	944 (30.1%)	426 (56.8%)	
Ventilator, No. (%)				<0.001
No	861 (22.2%)	805 (25.7%)	56 (7.5%)	
Yes	3,021 (77.8%)	2,327 (74.3%)	694 (92.5%)	
Cardiac Surgery, No. (%)				<0.001
No	2,849 (73.4%)	2,475 (79.0%)	374 (49.9%)	
Yes	1,033 (26.6%)	657 (21.0%)	376 (50.1%)	
Vital Signs				
HR, bpm	82 (72, 94)	82 (72, 95)	80 (72, 91)	0.033
MBP, mmHg	88 (76, 101)	89 (78, 102)	83 (72, 95)	<0.001
RR, bpm	18.0 (15.0, 22.0)	18.0 (15.0, 22.0)	17.0 (15.0, 21.0)	<0.001
Laboratory Parameters				
WBC, ×10³/μL	10.9 (8.1, 14.4)	10.8 (8.0, 14.3)	11.4 (8.5, 14.7)	0.015
RBC, ×10^6^/μL	3.92 (3.32, 4.47)	3.99 (3.43, 4.51)	3.58 (3.01, 4.14)	<0.001
Platelet, ×10³/μL	202 (151, 259)	209 (158, 264)	175 (127, 236)	<0.001
Calcium, mg/dL	8.60 (8.10, 9.00)	8.60 (8.10, 9.02)	8.40 (8.00, 8.80)	<0.001
Potassium, mEq/L	4.10 (3.80, 4.50)	4.10 (3.80, 4.50)	4.20 (3.80, 4.50)	0.007
Sodium, mEq/L	139.0 (136.0, 141.0)	139.0 (136.0, 141.0)	139.0 (136.0, 141.0)	0.012
Creatinine, mg/dL	0.90 (0.70, 1.20)	0.90 (0.70, 1.20)	1.00 (0.80, 1.30)	0.007
Comorbidities, No. (%)				
CHF	1,075 (27.7%)	760 (24.3%)	315 (42.0%)	<0.001
DM	1,434 (36.9%)	1,181 (37.7%)	253 (33.7%)	0.043
Glycemic Parameters				
HGI	0.81 (0.41, 1.77)	0.81 (0.41, 1.85)	0.83 (0.51, 1.47)	0.686
HGI group				<0.001
Q1	971 (25.0%)	829 (26.5%)	142 (18.9%)	
Q2	968 (24.9%)	748 (23.9%)	220 (29.3%)	
Q3	972 (25.0%)	735 (23.5%)	237 (31.6%)	
Q4	971 (25.0%)	820 (26.2%)	151 (20.1%)	
SHR	0.058 (0.048, 0.071)	0.058 (0.049, 0.072)	0.055 (0.047, 0.069)	<0.001
SHR group				0.001
Q1	970 (25.0%)	745 (23.8%)	225 (30.0%)	
Q2	970 (25.0%)	778 (24.8%)	192 (25.6%)	
Q3	970 (25.0%)	801 (25.6%)	169 (22.5%)	
Q4	972 (25.0%)	808 (25.8%)	164 (21.9%)	

Data are presented as medians (interquartile ranges) for continuous variables or numbers (%) for categorical variables. P values were compared between patients with and without NOAF via Mann–Whitney U tests for continuous variables and chi-square tests or Fisher’s exact tests for categorical variables, as appropriate. The quartiles (Q1–Q4) for HGI and SHR are defined by the distribution in the overall cohort. Abbreviations: BMI, body mass index; CCI, Charlson comorbidity index; CHF, congestive heart failure; CRRT, continuous renal replacement therapy; DM, diabetes mellitus; HGI, hemoglobin glycation index; HR, heart rate; MBP, mean blood pressure; NOAF, new-onset atrial fibrillation; RBC, red blood cell; RR, respiratory rate; SAPS II, simplified acute physiology score II; SOFA, Sequential Organ Failure Assessment; WBC, white blood cell; SHR, stress hyperglycemia ratio.

To investigate the relationship between glycemic variability and clinical outcomes, patients were stratified by HGI and SHR quartiles, revealing distinct association patterns. In the HGI group, Q4 was associated with greater chronic disease burden (higher CCI, diabetes incidence), whereas Q3 presented the highest acute illness severity (SAPS II) (**Supplementary Table S2**). Interestingly, in the SHR group, Q1 had the most severe acute conditions, with the highest SAPS II and SOFA scores, greater use of CRRT and vasopressors, and more cardiovascular complications (**Supplementary Table S3**).

### Cumulative incidence of NOAF according to HGI and SHR

**Figure 2:**
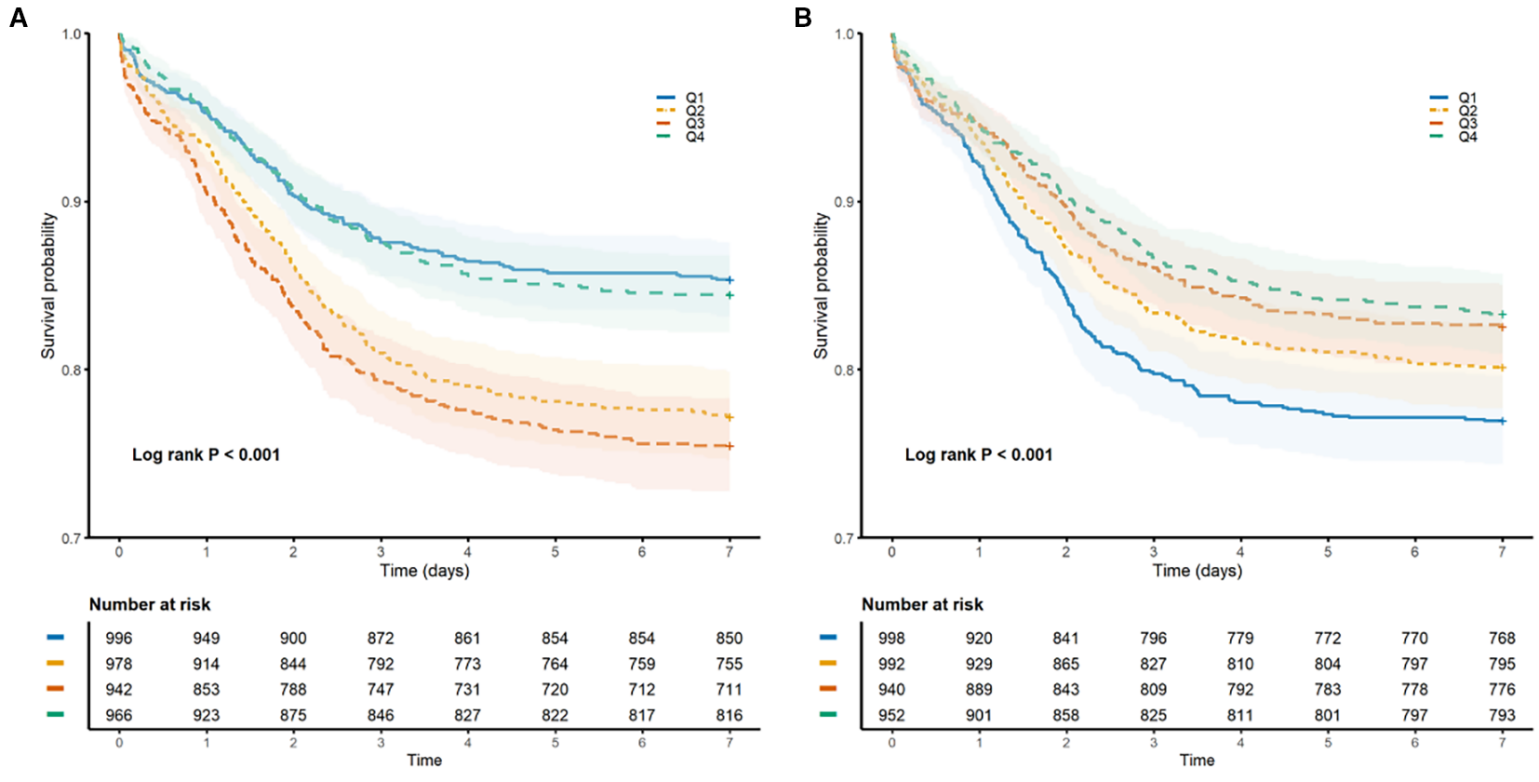
Kaplan–Meier curves illustrating the cumulative incidence of NOAF within 7 days of ICU admission, stratified by quartiles of glycemic indices. **(A)** Comparison across quartiles of the hemoglobin glycation index (HGI). **(B)** Comparisons across quartiles of the stress/hyperglycemia ratio (SHR). The vertical axis indicates the cumulative incidence rate; the horizontal axis indicates days since ICU admission.

During the seven-day follow-up period, 750 patients (19.3%) presented with NOAF. A Kaplan–Meier analysis revealed significant disparities in the cumulative incidence of NOAF across the quartiles of both the HGI and the SHR (log-rank test, both P < 0.001; **Figure 2**). For HGI, the highest cumulative incidence was found in the third quartile (Q3), followed by the second quartile (Q2), suggesting a nonlinear, inverted U-shaped relationship. In contrast, SHR demonstrated a consistent linear inverse pattern, such that the lowest quartile (Q1) was associated with the highest risk of NOAF, with a progressive linear decrease in incidence across quartiles.

### Associations between HGI or SHR and the risk of NOAF

To further investigate the relationships among HGI, SHR, and the risk of NOAF, we constructed multivariate Cox proportional hazards models. The results of the associations between quartiles of HGI and SHR with NOAF are presented in **Table 2**.

**Table 2:** Multivariate Cox regression analysis of NOAF risk according to quartiles of the hemoglobin glycation index (HGI) and stress/hyperglycemia ratio (SHR).

Outcomes exposure	Model 1		Model 2		Model 3	
	HR (95% CI)	*P*	HR (95% CI)	*P*	HR (95% CI)	*P*
HGI group						
Q1	Ref		Ref		Ref	
Q2	1.62 (1.31-2.00)	<0.001	1.40 (1.14-1.74)	0.002	1.26 (1.02-1.57)	0.034
Q3	1.78 (1.44-2.19)	<0.001	1.52 (1.23-1.88)	<0.001	1.36 (1.09-1.70)	0.006
Q4	1.06 (0.85-1.34)	0.602	1.14 (0.90-1.43)	0.282	1.28 (0.95-1.72)	0.102
SHR group						
Q1	Ref		Ref		Ref	
Q2	0.83 (0.68-1.01)	0.058	0.80 (0.66-0.97)	0.023	0.83 (0.68-1.01)	0.058
Q3	0.72 (0.59-0.88)	0.001	0.70 (0.57-0.85)	<0.001	0.76 (0.62-0.93)	0.007
Q4	0.69 (0.57-0.85)	<0.001	0.71 (0.58-0.86)	<0.001	0.69 (0.55-0.85)	<0.001

The data are presented as hazard ratios (HRs) and 95% confidence intervals (CIs).

Model 1: unadjusted.

Model 2: adjusted for age, sex, BMI, and race.

Model 3: additionally adjusted for SOFA score, SAPS II score, ventilator use, CRRT, vasopressor use, vital signs (HR, MBP, RR, SpO2), laboratory parameters (WBC, RBC, platelet, chloride, calcium, potassium, sodium, creatinine), comorbidities (MI, CHF, DM, AKI, delirium, renal disease, liver disease), and cardiac surgery.

For HGI, in the unadjusted (Model 1) and partially adjusted (Model 2) models, both the second (Q2) and third (Q3) quartiles were significantly associated with an increased risk of NOAF compared with the reference group (Q1). In the fully adjusted Model 3, HGI Q2 (HR, 1.26; 95% CI, 1.02–1.57; P = 0.034) and Q3 (HR, 1.36; 95% CI, 1.09–1.70; P = 0.006) remained independent risk factors for NOAF. Notably, the risk associated with the highest quartile (Q4) was not statistically significant after full multivariable adjustment (HR, 1.28; 95% CI, 0.95–1.72; P = 0.102).

For SHRs, a negative association with the risk of NOAF was consistently observed. In both the unadjusted (Model 1) and partially adjusted (Model 2) models, the third (Q3) and fourth (Q4) quartiles of SHR were associated with a significantly lower risk of NOAF than the lowest quartile (Q1). This association remained robust even after full adjustment in Model 3, where Q3 (HR, 0.76; 95% CI, 0.62–0.93; P = 0.007) and Q4 (HR, 0.69; 95% CI, 0.55–0.85; P < 0.001) were still linked to a significantly reduced risk of developing NOAF.

### Nonlinear and linear associations of HGI and SHR with the risk of NOAF

**Figure 3:**
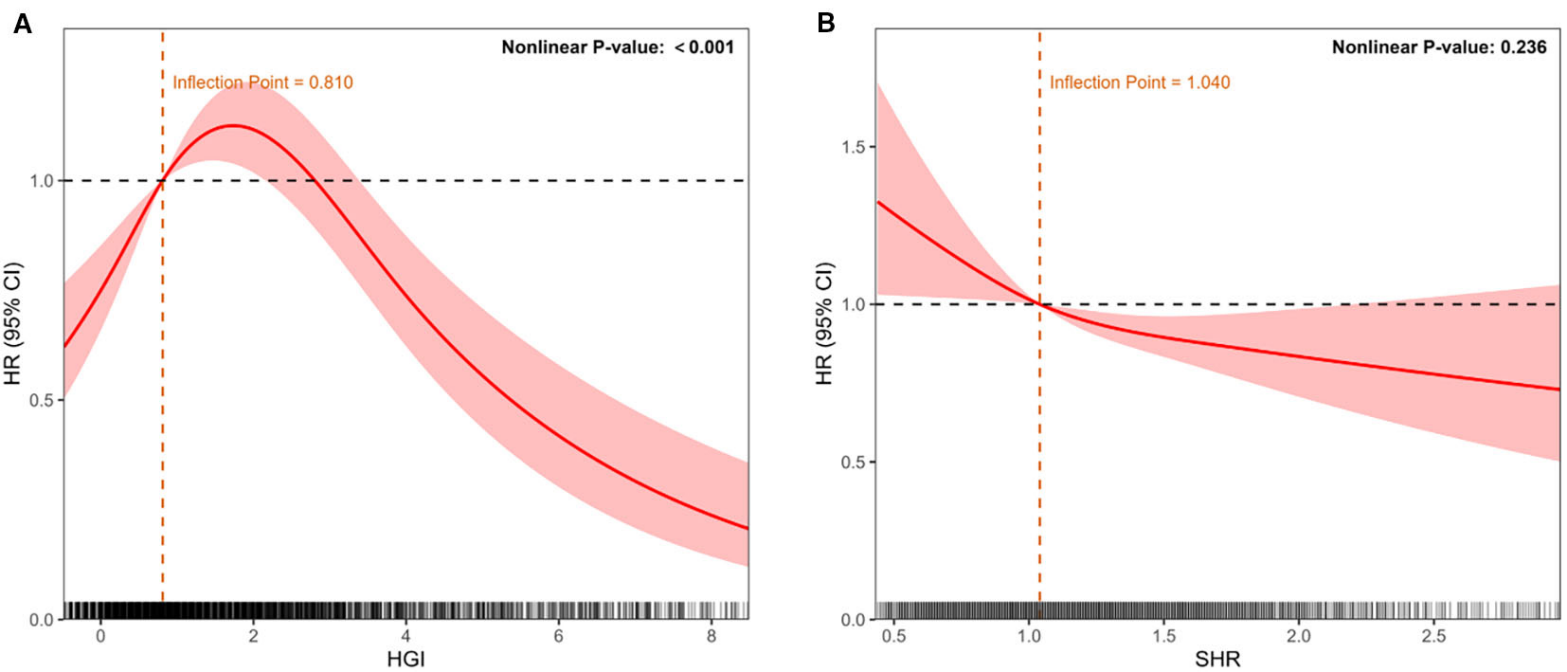
Restricted cubic spline models depicting continuous associations between glycemic indices and NOAF risk after adjustment for potential confounders (age, sex, race, BMI, SOFA score, SAPS II score, clinical interventions, laboratory parameters, and comorbidities). **(A)** Association of HGI with NOAF, demonstrating an inverted U-shaped relationship. **(B)** Association of the stress–hyperglycemia ratio (SHR) with NOAF, demonstrating a linear inverse relationship. The solid red line represents the adjusted hazard ratio (HR); shaded areas indicate 95% confidence intervals (CIs). The dashed line indicates HR = 1. The histograms below depict the data distribution.

Using restricted cubic spline (RCS) models, we evaluated the continuous associations between HGI, SHR, and the risk of NOAF, adjusting for the predefined covariate set used in Model 3. As illustrated in **Figure 3A**, HGI showed a significant inverted U−shaped relationship with NOAF (P for nonlinearity < 0.001), with risk peaking at mid−range values and attenuating toward both tails. As shown in **Figure 3B**, SHR displayed a predominantly linear inverse association (P for nonlinearity = 0.236), indicating a progressively lower NOAF risk at higher SHR levels. These spline patterns are concordant with the quartile analyses (higher risk in HGI Q2–Q3 vs Q1; lower risk across higher SHR quartiles) and avoid reliance on arbitrary cutoff points.

### Subgroup analyses

**Figure 4:**
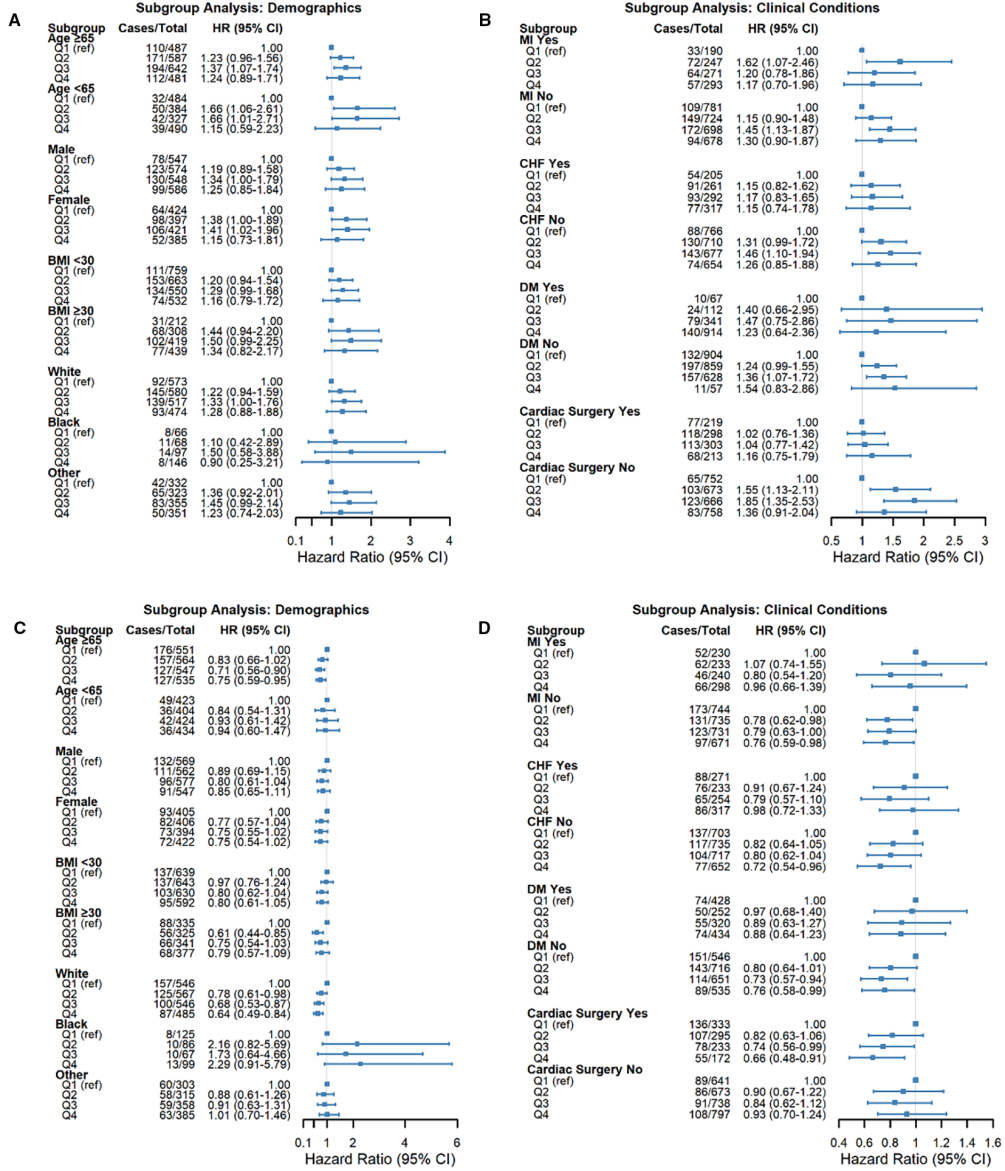
Forest plots demonstrating subgroup analyses for the associations between glycemic indices and NOAF risk. The subgroups included age, sex, BMI, race, myocardial infarction (MI), congestive heart failure (CHF), diabetes mellitus (DM), and cardiac surgery history. **(A, B)** Associations of HGI with NOAF risk across specified subgroups. **(C, D)** Associations of the stress/hyperglycemia ratio (SHR) with NOAF risk across specified subgroups. Squares represent hazard ratios (HRs), and horizontal lines represent 95% confidence intervals (CIs). The vertical dashed line (HR = 1.0) indicates no effect. P values indicate significant interactions between subgroups.

We performed prespecified risk subgroup analyses and multiplicative interaction tests according to routinely reported strata: age (e.g., <65 vs. ≥65 years), sex, BMI (e.g., <30 vs. ≥30 kg/m²), race (white, black, and other), and clinical history (MI, CHF, DM, prior cardiac surgery), using the same covariate set as Model 3. **Figure 4** presents the adjusted hazard ratios with 95% CIs for each stratum and the corresponding P values for interaction.

For HGI, the association with NOAF was directionally consistent across strata, with the excess risk concentrated in mid−range categories (Q2–Q3 vs Q1) in multiple groups (e.g., females, individuals aged ≥65 years, and patients without prior MI/CHF/DM). When sample sizes were smaller, CIs widened, and statistical significance varied, but the pattern of a mid−range elevation was preserved.

For SHRs, higher quartiles were generally associated with lower NOAF risk, mirroring the main analysis. The inverse gradient was most evident in clinically common strata (e.g., older adults, White patients, and those without MI/CHF/DM or with prior cardiac surgery), whereas some subgroups presented wider CIs and non−significant estimates.

Crucially, no effect modification was detected—all P values for interaction > 0.05 for both HGI and SHR—indicating that the observed subgroup differences are descriptive rather than confirmatory. Accordingly, the pooled estimates provide the most reliable summary of the associations, and subgroup findings should be interpreted with caution given the varying precision across strata.

### Diabetes-stratified subgroup analysis

We observed distinct patterns according to diabetes status (**Supplementary Figure S1**, **Supplementary Table S3**). Among nondiabetic patients, higher HGI quartiles were associated with increased NOAF risk after full adjustment (Q3 vs Q1: HR 1.37, 95% CI 1.04–1.81, P = 0.024; Q4 vs Q1: HR 1.37, 95% CI 1.04–1.81, P = 0.024). Conversely, a higher SHR was protective (Q3 vs. Q1: HR 0.75, 95% CI 0.58–0.96, P = 0.024; Q4 vs. Q1: HR 0.67, 95% CI 0.51–0.87, P = 0.003). Among diabetic patients, HGI quartile was not significantly associated with NOAF; for SHRs, only Q4 was associated with a lower risk (HR 0.64, 95% CI 0.43–0.94; P = 0.024). Where available, P for the exposure × diabetes interaction is shown in **Figure 4** (numerical values in **Supplementary Table S3**).

## Discussion

This study elucidates the relationship between HGI/SHR and short−term arrhythmic outcomes in critically ill adults. To our knowledge, this is the first ICU investigation to evaluate these indices side−by−side for the 7−day incidence of NOAF. The principal findings were as follows: (1) HGI exhibited a nonlinear, inverted−U association with NOAF; versus Q1, risk was higher in Q2–Q3 by approximately 25–36%, whereas Q4 was not materially different. (2) SHR was inversely associated with NOAF, with a lower risk in Q3–Q4 by approximately 24–31% than in Q1. (3) Restricted cubic spline analyses corroborated these patterns, demonstrating strong nonlinearity for HGI (P for nonlinearity < 0.001), with the risk peak in the middle−range of HGI, and no evidence of nonlinearity for SHR (P for nonlinearity = 0.236. (4) The results were broadly consistent across predefined subgroups (all P values for interaction > 0.05). (5) In diabetes−stratified analyses, associations were more pronounced among nondiabetic patients, whereas in those with diabetes, only the highest SHR quartile (Q4) retained a protective association. Collectively, these covariate−adjusted patterns suggest that a mismatch between acute and chronic glycemia may be most arrhythmogenic at intermediate HGI, whereas low SHR may reflect a blunted stress−glycemic response; these interpretations remain hypothesis−generating.

Prior investigations have linked the hemoglobin glycation index (HGI) to major adverse cardiovascular events, heart failure progression, and all-cause mortality across general and cardiovascular cohorts, most often describing U- or J-shaped associations (26–28). The SHR has likewise been associated with short- and long-term mortality in acute coronary syndrome patients and acute heart failure patients and with perioperative complications—including postoperative atrial fibrillation—in cardiac-surgical populations (29–33). In intensive care unit (ICU) settings, the SHR and conceptually related “mismatch” indices are correlated with adverse outcomes among patients with sepsis and cardio-cerebrovascular disease (34, 35). However, evidence specific to incident NOAF in heterogeneous ICU cohorts remains limited, and reported association shapes vary by endpoint, population, and exposure.

Against this backdrop, our study extends prior work by (i) evaluating HGI and SHR side-by-side for 7-day NOAF and (ii) characterizing dose–response shapes rather than assuming linearity, showing an inverted-U pattern for HGI and an approximately linear inverse association for SHR—these findings are consistent with the notion that these indices capture distinct glycemic phenotypes with differential relevance to arrhythmogenesis in critical illness.

The association between glycemic indices and early NOAF in critical illness is biologically plausible (15, 25). Higher glycation propensity has been linked to AGE–RAGE activation, oxidative stress, endothelial dysfunction, and low−grade inflammation, processes that may contribute to atrial substrate remodeling (fibrosis, conduction heterogeneity) and ion−handling abnormalities predisposed to triggered activity (36–40). Within this framework, our inverted−U pattern for HGI may indicate that arrhythmic risk peaks when there is a pronounced mismatch between acute and chronic glycemia—sufficient to amplify inflammatory and autonomic perturbations—yet without the longer−term adaptations observed at very high glycation burdens. In the ICU, additional stressors (hemodynamic instability, hypoxemia, electrolyte derangements, and catecholaminergic fluctuations) could further lower the threshold for atrial ectopy and re−entry (41–43).

In contrast, the approximately linear inverse relationship between SHR and NOAF suggests that a low SHR may indicate a blunted counterregulatory response or limited metabolic reserve (e.g., impaired β−cell output, mitochondrial energetics, or adrenal/adrenergic insufficiency), a state often accompanied by hypotension and greater vasopressor requirements that can favor arrhythmogenesis (44, 45). This interpretation may also help reconcile the shape differences reported for ischemic endpoints (where higher stress hyperglycemia is frequently harmful) versus arrhythmic outcomes, which depend more on autonomic balance, substrate vulnerability, and conduction properties than on atherosclerotic burden per se (45–48). These explanations are hypothesis−generating and could be influenced by residual confounding or reverse causation. Prospective studies incorporating continuous glucose and rhythm monitoring, together with biomarker panels (e.g., AGEs/sRAGE, hs−CRP, and IL−6) and autonomic indices, will be needed to validate or refute these mechanisms.

In addition to metabolic and inflammatory pathways, dysglycemia may also operate through a neuro-cardiac axis (49). AGEs and receptors for AGE (RAGE)-mediated injury to peripheral and autonomic nerves can perturb the autonomic balance and precipitate atrial ectopy (49–52). The related concept of “type 3 diabetes”—brain insulin resistance—underscores the systemic scope of this pathway (53). These mechanisms were not directly assessed or phenotyped in the present study and should be considered hypothesis-generating.

The subgroup and sensitivity analyses were directionally consistent yet clinically informative. In nondiabetic patients, the covariate-adjusted associations were more pronounced—intermediate HGI was associated with higher NOAF risk and higher SHR with lower risk—whereas among patients with diabetes, the inverse SHR signal was mainly evident at the highest quartile, and HGI quartiles were not clearly differentiated. Across other prespecified strata (age, sex, BMI, race, prior myocardial infarction or heart failure, and prior cardiac surgery), we found no statistically significant interactions (all P values for interactions > 0.05), supporting broad applicability. Taken together, this pattern suggests that the baseline glycemic milieu may modulate how acute glycemic responses are related to arrhythmic vulnerability—consistent with the notion that, in the absence of chronic hyperglycemic adaptation, acute glycemic dysregulation may exert greater proarrhythmic effects. These inferences are hypothesis-generating and may be influenced by residual confounding or differential power across strata. Interindividual susceptibility likely varies by genetics: canonical atrial fibrillation loci (e.g., PITX2, ZFHX3) and variants in glycation pathway genes (e.g., AGER) may modify the association between acute–chronic glycemic mismatch and new-onset atrial fibrillation (NOAF) (54, 55). Rigorous evaluation will require multiocestry cohorts with genotyping to test gene–glycemia interactions and effect modifications.

These results are best used to inform monitoring rather than to define therapeutic targets. We propose a pragmatic bundle for exploratory high-risk zones identified by cohort-derived quartiles: intermediate HGI (Q2–Q3) and low SHR (Q1). For such patients, we suggest (i) continuous telemetry during the first 7 days (or ICU stay, whichever is longer); (ii) daily electrolytes with proactive maintenance (e.g., K 4.0–4.5 mEq/L, Mg ≥ 2.0 mg/dL) to reduce ectopy; (iii) capillary glucose checks per ICU protocol to avoid hypoglycemia and large glycemic excursions; and (iv) early 12-lead ECG for symptoms or monitor alerts. These steps are nonprescriptive and intended to support hypothesis testing; they will require prospective validation and local adaptation. From a postdischarge, translational standpoint, structured, coach-led diabetes programs warrant testing as a strategy to improve ambulatory glycemic control and, in turn, to assess whether arrhythmic risk can be mitigated. Successful adoption will depend on appropriate patient selection, the choice of delivery modality (e.g., telehealth vs. in person), and seamless integration with routine cardiology follow-up.

This study has several strengths. First, it leverages a large, well-characterized ICU cohort and a prespecified analytic framework incorporating quartile-based contrasts, restricted cubic spline modeling, predefined subgroup and interaction testing, and diabetes-stratified sensitivity analyses. Second, extensive covariate adjustment was undertaken to mitigate confounding.

Nevertheless, several limitations merit consideration. The retrospective, single-center design limits generalizability and precludes causal inference. Residual confounding remains possible, particularly from unmeasured inflammatory or autonomic markers. Rhythm ascertainment relies on clinical documentation, which may introduce misclassification, and exposure–outcome temporality may be imperfect despite early capture windows. The pathophysiological interpretations are therefore hypothesis-generating and require prospective validation. Finally, neuropathy, autonomic function indices, cognition, and genetic variation were not assessed; accordingly, the proposed neuro–cardiac and gene–environment mechanisms remain speculative.

## Conclusions

In conclusion, this retrospective cohort study of critically ill adults admitted to the ICU revealed that HGI and SHR—two readily obtainable indices integrating admission glucose with chronic glycemic background—exhibited differential, covariate−adjusted associations with 7−day incident NOAF: an inverted−U pattern for HGI and an approximately linear inverse pattern for SHR. When used alongside admission glucose and HbA1c, these measures may enhance short−term risk stratification and inform rhythm surveillance without implying therapeutic thresholds. Nevertheless, given the single−center, observational design, these findings require external validation. Prospective, multicenter investigations incorporating standardized rhythm monitoring, continuous glucose profiling, and biomarker assessments are needed to confirm generalizability, clarify mechanisms, and evaluate clinical utility.

## Data Availability

The raw data supporting the conclusions of this article will be made available by the authors, without undue reservation.

## Supplementary material

Supplementary Table 1 Additional baseline characteristics of critically ill patients with and without new-onset atrial fibrillation (NOAF).

Supplementary Table 2 Baseline demographics and clinical characteristics stratified by quartiles of the hemoglobin glycation index (HGI). Data are presented as medians (interquartile ranges) for continuous variables or numbers (%) for categorical variables. Differences across quartiles were assessed via the Kruskal–Wallis test or chi-square test, as appropriate. Abbreviations are listed in **Table 1**.

Supplementary Table 3 Baseline demographics and clinical characteristics stratified by quartiles of the stress–hyperglycemia ratio (SHR). Data are presented as medians (interquartile ranges) for continuous variables or numbers (%) for categorical variables. Differences across quartiles were assessed via the Kruskal–Wallis test or chi-square test, as appropriate. Abbreviations are listed in **Table 1**.

Supplementary Table 4 Subgroup analyses for the associations between glycemic indices and incident NOAF within 7 days after ICU admission. Data are presented as hazard ratios (HRs) with 95% confidence intervals (CIs). Model 1: unadjusted. Model 2: adjusted for age, sex, BMI, and race. Model 3: additionally adjusted for SOFA score, SAPS II score, ventilator use, CRRT, vasopressor use, vital signs (HR, MBP, RR, SpO2), laboratory parameters (WBC, RBC, platelet, chloride, calcium, potassium, sodium, creatinine), comorbidities (MI, CHF, DM, AKI, delirium, renal disease, liver disease), and cardiac surgery.

Supplementary Figure 1 Associations of the hemoglobin glycation index (HGI) and stress hyperglycemia ratio (SHR) with new-onset atrial fibrillation (NOAF), stratified by diabetes status (DM vs. non-DM), across three Cox regression models. Hazard ratios (HRs) are shown for quartiles of each glycemic index, with the lowest quartile (Q1) as a reference: **(A)** patients with diabetes (DM) and **(B)** patients without diabetes (non-DM). Within each panel, the results are presented separately for HGI (top) and SHR (bottom) under Models 1–3 (definitions and covariate adjustments provided in the Methods).
