# Supplementary material for: Distinct patterns of association between the hemoglobin glycation index, the stress–hyperglycemia ratio, and the risk of new-onset atrial fibrillation in critically ill patients
Source: Front Endocrinol (Lausanne). 2025 Sep 17;16:1656783. doi: 10.3389/fendo.2025.1656783 (PMC12483897; doi:10.3389/fendo.2025.1656783)

**Supplementary material**

**Table S1.** Additional baseline characteristics of critically ill patients with and without new-onset atrial fibrillation (NOAF)

|  |  | **NOAF** |  |  |
| --- | --- | --- | --- | --- |
| **Characteristic** | **Overall  N = 3,882** | **No  N = 3,132** | **Yes  N = 750** | **p value** |
| **Vital Signs** | | | | |
| SBP, mmHg | 129 (112, 147) | 130 (114, 148) | 121 (106, 140) | <0.001 |
| DBP, mmHg | 70 (60, 84) | 72 (61, 85) | 65 (55, 76) | <0.001 |
| Temperature, °C | 36.72 (36.44, 37.00) | 36.72 (36.44, 37.06) | 36.55 (36.11, 36.89) | <0.001 |
| SpO2, % | 98.00 (96.00, 100.00) | 98.00 (96.00, 100.00) | 99.00 (96.00, 100.00) | 0.002 |
| **Laboratory Parameters** | | | | |
| Glucose, mg/dL | 128 (104, 177) | 130 (105, 180) | 122 (101, 160) | <0.001 |
| Chloride, mEq/L | 104.0 (101.0, 107.0) | 104.0 (101.0, 107.0) | 106.0 (101.0, 109.0) | <0.001 |
| **Comorbidities, No. (%)** | | | | |
| MI | 1,001 (25.8%) | 775 (24.7%) | 226 (30.1%) | 0.002 |
| AKI | 3,266 (84.1%) | 2,567 (82.0%) | 699 (93.2%) | <0.001 |
| Delirium | 789 (20.3%) | 638 (20.4%) | 151 (20.1%) | 0.885 |
| Renal Disease | 678 (17.5%) | 506 (16.2%) | 172 (22.9%) | <0.001 |
| Malignant Cancer | 214 (5.5%) | 184 (5.9%) | 30 (4.0%) | 0.043 |
| Liver Disease | 271 (7.0%) | 235 (7.5%) | 36 (4.8%) | 0.009 |

**Table S2. Baseline demographics and clinical characteristics stratified by quartiles of the hemoglobin glycation index (HGI)**

|  |  |  | **HGIgroup** |  |  |  |
| --- | --- | --- | --- | --- | --- | --- |
| Characteristic | **Overall  N = 3,882** | **Q1 N = 971** | **Q2  N = 968** | **Q3  N = 972** | **Q4  N = 971** | **p value** |
| Age, Median (Q1, Q3) | 68 (57, 77) | 65 (53, 76) | 69 (59, 79) | 71 (61, 80) | 65 (55, 75) | <0.001 |
| Gender, n (%) |  |  |  |  |  | 0.206 |
| Female | 1,627 (41.9%) | 424 (43.7%) | 396 (40.9%) | 422 (43.4%) | 385 (39.6%) |  |
| Male | 2,255 (58.1%) | 547 (56.3%) | 572 (59.1%) | 550 (56.6%) | 586 (60.4%) |  |
| Race, n (%) |  |  |  |  |  | <0.001 |
| Black | 377 (9.7%) | 66 (6.8%) | 68 (7.0%) | 97 (10.0%) | 146 (15.0%) |  |
| Other | 1,361 (35.1%) | 332 (34.2%) | 321 (33.2%) | 357 (36.7%) | 351 (36.1%) |  |
| White | 2,144 (55.2%) | 573 (59.0%) | 579 (59.8%) | 518 (53.3%) | 474 (48.8%) |  |
| BMI, Median (Q1, Q3) | 28.9 (26.2, 31.2) | 28.1 (25.5, 29.7) | 28.7 (25.6, 30.6) | 29.4 (26.8, 32.1) | 29.6 (26.7, 32.2) | <0.001 |
| SOFA, Median (Q1, Q3) | 1.00 (0.00, 2.00) | 1.00 (0.00, 2.00) | 1.00 (0.00, 2.00) | 1.00 (0.00, 3.00) | 1.00 (0.00, 2.00) | 0.110 |
| SAPS_II, Median (Q1, Q3) | 33 (25, 41) | 31 (24, 40) | 32 (25, 41) | 34 (27, 42) | 33 (25, 42) | <0.001 |
| CCI, Median (Q1, Q3) | 5.00 (3.00, 7.00) | 4.00 (3.00, 7.00) | 5.00 (3.00, 7.00) | 5.00 (4.00, 7.00) | 6.00 (4.00, 8.00) | <0.001 |
| Ventilator, n (%) | 3,021 (77.8%) | 712 (73.3%) | 760 (78.5%) | 805 (82.8%) | 744 (76.6%) | <0.001 |
| No | 861 (22.2%) | 259 (26.7%) | 208 (21.5%) | 167 (17.2%) | 227 (23.4%) |  |
| Yes | 3,021 (77.8%) | 712 (73.3%) | 760 (78.5%) | 805 (82.8%) | 744 (76.6%) |  |
| CRRT, n (%) | 148 (3.8%) | 43 (4.4%) | 35 (3.6%) | 28 (2.9%) | 42 (4.3%) | 0.250 |
| No | 3,734 (96.2%) | 928 (95.6%) | 933 (96.4%) | 944 (97.1%) | 929 (95.7%) |  |
| Yes | 148 (3.8%) | 43 (4.4%) | 35 (3.6%) | 28 (2.9%) | 42 (4.3%) |  |
| Vasopressor, n (%) | 1,370 (35.3%) | 298 (30.7%) | 353 (36.5%) | 402 (41.4%) | 317 (32.6%) | <0.001 |
| No | 2,512 (64.7%) | 673 (69.3%) | 615 (63.5%) | 570 (58.6%) | 654 (67.4%) |  |
| Yes | 1,370 (35.3%) | 298 (30.7%) | 353 (36.5%) | 402 (41.4%) | 317 (32.6%) |  |
| Cardiac_Surgery, n (%) | 1,033 (26.6%) | 219 (22.6%) | 297 (30.7%) | 304 (31.3%) | 213 (21.9%) | <0.001 |
| No | 2,849 (73.4%) | 752 (77.4%) | 671 (69.3%) | 668 (68.7%) | 758 (78.1%) |  |
| Yes | 1,033 (26.6%) | 219 (22.6%) | 297 (30.7%) | 304 (31.3%) | 213 (21.9%) |  |
| HR, Median (Q1, Q3) | 82 (72, 94) | 82 (72, 94) | 80 (70, 89) | 81 (71, 93) | 87 (75, 100) | <0.001 |
| SBP, Median (Q1, Q3) | 129 (112, 147) | 127 (112, 143) | 129 (111, 145) | 128 (112, 147) | 131 (113, 150) | 0.015 |
| DBP, Median (Q1, Q3) | 70 (60, 84) | 71 (61, 85) | 71 (60, 84) | 69 (58, 83) | 70 (59, 83) | 0.073 |
| MBP, Median (Q1, Q3) | 88 (76, 101) | 88 (77, 101) | 88 (77, 101) | 87 (76, 101) | 88 (76, 101) | 0.742 |
| RR, Median (Q1, Q3) | 18.0 (15.0, 22.0) | 18.0 (15.0, 21.2) | 18.0 (15.0, 21.0) | 18.0 (15.0, 21.0) | 19.0 (16.0, 23.0) | <0.001 |
| Temperature, Median (Q1, Q3) | 36.72 (36.44, 37.00) | 36.72 (36.44, 37.06) | 36.67 (36.33, 36.94) | 36.67 (36.41, 37.00) | 36.78 (36.50, 37.06) | <0.001 |
| SpO2, Median (Q1, Q3) | 98.00 (96.00, 100.00) | 98.00 (96.00, 100.00) | 98.00 (96.00, 100.00) | 98.00 (95.00, 100.00) | 98.00 (96.00, 100.00) | 0.032 |
| HbA1c, Median (Q1, Q3) | 5.80 (5.40, 6.80) | 5.20 (5.00, 5.30) | 5.60 (5.50, 5.70) | 6.10 (6.00, 6.30) | 8.40 (7.40, 10.40) | <0.001 |
| WBC, Median (Q1, Q3) | 10.9 (8.1, 14.4) | 10.7 (7.9, 14.3) | 10.7 (8.0, 13.9) | 11.0 (8.1, 14.4) | 11.1 (8.4, 14.9) | 0.023 |
| RBC, Median (Q1, Q3) | 3.92 (3.32, 4.47) | 3.78 (3.18, 4.34) | 3.96 (3.34, 4.47) | 3.93 (3.30, 4.53) | 3.99 (3.49, 4.51) | <0.001 |
| Platelet, Median (Q1, Q3) | 202 (151, 259) | 192 (144, 251) | 199 (146, 249) | 201 (152, 259) | 218 (163, 273) | <0.001 |
| Glucose, Median (Q1, Q3) | 7.1 (5.8, 9.8) | 6.2 (5.4, 7.4) | 6.4 (5.6, 7.8) | 7.1 (5.8, 8.9) | 11.7 (8.4, 16.3) | <0.001 |
| Chloride, Median (Q1, Q3) | 104.0 (101.0, 107.0) | 105.0 (101.0, 108.0) | 105.0 (102.0, 108.0) | 104.0 (101.0, 108.0) | 103.0 (99.0, 106.0) | <0.001 |
| Calcium, Median (Q1, Q3) | 8.60 (8.10, 9.00) | 8.60 (8.10, 9.00) | 8.60 (8.10, 9.00) | 8.60 (8.10, 9.00) | 8.60 (8.10, 9.10) | 0.436 |
| Potassium, Median (Q1, Q3) | 4.10 (3.80, 4.50) | 4.00 (3.70, 4.40) | 4.10 (3.75, 4.40) | 4.10 (3.80, 4.50) | 4.20 (3.80, 4.60) | <0.001 |
| Sodium, Median (Q1, Q3) | 139.0 (136.0, 141.0) | 139.0 (136.0, 141.0) | 139.0 (137.0, 141.0) | 139.0 (136.0, 141.0) | 138.0 (135.0, 140.0) | <0.001 |
| Creatinine, Median (Q1, Q3) | 0.90 (0.70, 1.20) | 0.90 (0.70, 1.20) | 0.90 (0.70, 1.20) | 0.90 (0.70, 1.20) | 1.00 (0.80, 1.40) | <0.001 |
| NOAF, n (%) | 1,033 (26.6%) | 219 (22.6%) | 297 (30.7%) | 304 (31.3%) | 213 (21.9%) | <0.001 |
| MI, n (%) | 1,001 (25.8%) | 190 (19.6%) | 246 (25.4%) | 272 (28.0%) | 293 (30.2%) | <0.001 |
| CHF, n (%) | 1,075 (27.7%) | 205 (21.1%) | 260 (26.9%) | 293 (30.1%) | 317 (32.6%) | <0.001 |
| Dementia, n (%) | 144 (3.7%) | 43 (4.4%) | 33 (3.4%) | 29 (3.0%) | 39 (4.0%) | 0.342 |
| DM, n (%) | 1,434 (36.9%) | 67 (6.9%) | 112 (11.6%) | 341 (35.1%) | 914 (94.1%) | <0.001 |
| AKI, n (%) | 3,266 (84.1%) | 802 (82.6%) | 817 (84.4%) | 859 (88.4%) | 788 (81.2%) | <0.001 |
| Renal Disease, n (%) | 678 (17.5%) | 137 (14.1%) | 130 (13.4%) | 190 (19.5%) | 221 (22.8%) | <0.001 |
| Liver Disease, n (%) | 271 (7.0%) | 94 (9.7%) | 52 (5.4%) | 72 (7.4%) | 53 (5.5%) | <0.001 |

Data are presented as medians (interquartile ranges) for continuous variables or numbers (%) for categorical variables. Differences across quartiles were assessed via the Kruskal–Wallis test or chi-square test, as appropriate. Abbreviations are listed in Table 1.

**Table S3. Baseline demographics and clinical characteristics stratified by quartiles of the stress–hyperglycemia ratio (SHR)**

|  |  |  | **SHRgroup** |  |  |  |
| --- | --- | --- | --- | --- | --- | --- |
| **Characteristic** | **Overall  N = 3,882** | **Q1 N = 920** | **Q2  N = 929** | **Q3  N = 940** | **Q4  N = 952** | **p value** |
| Age, Median (Q1, Q3) | 68 (57, 77) | 67 (57, 76) | 68 (57, 78) | 68 (58, 78) | 68 (56, 77) | 0.221 |
| Gender, n (%) |  |  |  |  |  | 0.554 |
| Female | 1,627 (41.9%) | 424 (46.1%) | 404 (43.5%) | 407 (43.3%) | 392 (41.2%) |  |
| Male | 2,255 (58.1%) | 548 (59.6%) | 566 (60.9%) | 563 (59.9%) | 578 (60.7%) |  |
| Race, n (%) |  |  |  |  |  | <0.001 |
| White | 2,144 (55.2%) | 487 (52.9%) | 545 (58.7%) | 566 (60.2%) | 546 (57.4%) |  |
| Black | 377 (9.7%) | 99 (10.8%) | 125 (13.5%) | 86 (9.1%) | 67 (7.0%) |  |
| Other | 1,361 (35.1%) | 386 (42.0%) | 300 (32.3%) | 318 (33.8%) | 357 (37.5%) |  |
| BMI, Median (Q1, Q3) | 28.9 (26.2, 31.2) | 29.0 (25.7, 31.7) | 28.8 (25.7, 30.9) | 28.8 (26.7, 30.7) | 28.9 (26.3, 31.1) | 0.628 |
| SOFA, Median (Q1, Q3) | 1.00 (0.00, 2.00) | 1.00 (0.00, 3.00) | 1.00 (0.00, 2.00) | 1.00 (0.00, 2.00) | 1.00 (0.00, 2.00) | 0.010 |
| SAPS_II, Median (Q1, Q3) | 33 (25, 41) | 36 (29, 46) | 32 (25, 40) | 31 (24, 39) | 33 (24, 40) | <0.001 |
| CCI, Median (Q1, Q3) | 5.00 (3.00, 7.00) | 5.00 (4.00, 7.00) | 5.00 (3.00, 7.00) | 5.00 (3.00, 7.00) | 5.00 (3.00, 7.00) | 0.007 |
| Ventilator, n (%) | 3,021 (77.8%) | 823 (89.5%) | 724 (77.9%) | 723 (76.9%) | 751 (78.9%) | <0.001 |
| CRRT, n (%) | 148 (3.8%) | 71 (7.7%) | 19 (2.0%) | 26 (2.8%) | 32 (3.4%) | <0.001 |
| Vasopressor, n (%) | 1,370 (35.3%) | 399 (43.4%) | 363 (39.1%) | 310 (33.0%) | 298 (31.3%) | <0.001 |
| Cardiac Surgery, n (%) | 1,033 (26.6%) | 172 (18.7%) | 331 (35.6%) | 296 (31.5%) | 234 (24.6%) | <0.001 |
| HR, Median (Q1, Q3) | 82 (72, 94) | 87 (76, 101) | 80 (71, 90) | 80 (71, 90) | 82 (72, 94) | <0.001 |
| SBP, Median (Q1, Q3) | 129 (112, 147) | 128 (111, 146) | 129 (111, 147) | 128 (112, 147) | 131 (114, 147) | 0.359 |
| DBP, Median (Q1, Q3) | 70 (60, 84) | 69 (59, 84) | 70 (59, 82) | 71 (60, 85) | 71 (60, 83) | 0.329 |
| MBP, Median (Q1, Q3) | 88 (76, 101) | 86 (76, 101) | 88 (76, 100) | 88 (77, 103) | 88 (77, 100) | 0.212 |
| RR, Median (Q1, Q3) | 18.0 (15.0, 22.0) | 19.0 (16.0, 24.0) | 17.0 (15.0, 20.0) | 18.0 (15.0, 21.0) | 18.0 (15.0, 21.0) | <0.001 |
| Temperature, Median (Q1, Q3) | 36.72 (36.44, 37.00) | 36.67 (36.39, 37.00) | 36.67 (36.39, 37.00) | 36.72 (36.44, 37.00) | 36.72 (36.44, 37.06) | 0.015 |
| SpO2, Median (Q1, Q3) | 98.00 (96.00, 100.00) | 98.00 (96.00, 100.00) | 98.00 (96.00, 100.00) | 98.00 (96.00, 100.00) | 98.00 (96.00, 100.00) | 0.061 |
| HbA1c, Median (Q1, Q3) | 5.80 (5.40, 6.80) | 5.90 (5.40, 7.10) | 6.10 (5.70, 7.80) | 5.70 (5.40, 6.20) | 5.70 (5.30, 6.30) | <0.001 |
| WBC, Median (Q1, Q3) | 10.9 (8.1, 14.4) | 12.7 (9.4, 16.9) | 9.8 (7.2, 13.0) | 9.9 (7.6, 13.1) | 11.4 (9.0, 14.5) | <0.001 |
| RBC, Median (Q1, Q3) | 3.92 (3.32, 4.47) | 3.90 (3.29, 4.46) | 3.87 (3.27, 4.44) | 3.97 (3.36, 4.48) | 3.96 (3.39, 4.49) | 0.031 |
| Platelet, Median (Q1, Q3) | 202 (151, 259) | 208 (155, 269) | 199 (147, 254) | 199 (152, 251) | 204 (150, 261) | 0.034 |
| Glucose, Median (Q1, Q3) | 7.1 (5.8, 9.8) | 11.3 (9.0, 16.0) | 5.4 (4.9, 6.6) | 6.2 (5.7, 6.9) | 7.4 (6.7, 8.8) | <0.001 |
| Chloride, Median (Q1, Q3) | 104.0 (101.0, 107.0) | 103.0 (99.0, 107.0) | 105.0 (102.0, 108.0) | 105.0 (101.0, 108.0) | 104.0 (101.0, 107.0) | <0.001 |
| Calcium, Median (Q1, Q3) | 8.60 (8.10, 9.00) | 8.50 (8.00, 9.00) | 8.60 (8.10, 9.00) | 8.60 (8.12, 9.00) | 8.60 (8.11, 9.00) | 0.005 |
| Potassium, Median (Q1, Q3) | 4.10 (3.80, 4.50) | 4.20 (3.80, 4.60) | 4.10 (3.80, 4.50) | 4.10 (3.80, 4.40) | 4.10 (3.80, 4.50) | 0.002 |
| Sodium, Median (Q1, Q3) | 139.0 (136.0, 141.0) | 138.0 (135.0, 141.0) | 139.0 (137.0, 142.0) | 139.0 (137.0, 141.0) | 138.0 (136.0, 141.0) | <0.001 |
| Creatinine, Median (Q1, Q3) | 0.90 (0.70, 1.20) | 1.10 (0.80, 1.50) | 0.90 (0.70, 1.20) | 0.90 (0.70, 1.10) | 0.90 (0.70, 1.20) | <0.001 |
| MI, n (%) | 1,001 (25.8%) | 298 (32.4%) | 228 (24.5%) | 234 (24.9%) | 241 (25.3%) | <0.001 |
| CHF, n (%) | 1,075 (27.7%) | 318 (34.6%) | 271 (29.2%) | 233 (24.8%) | 253 (26.6%) | <0.001 |
| Dementia, n (%) | 144 (3.7%) | 38 (4.1%) | 36 (3.9%) | 33 (3.5%) | 37 (3.9%) | 0.941 |
| DM, n (%) | 1,434 (36.9%) | 434 (47.2%) | 427 (46.0%) | 253 (26.9%) | 320 (33.6%) | <0.001 |
| AKI, n (%) | 3,266 (84.1%) | 856 (93.0%) | 798 (85.9%) | 791 (84.1%) | 821 (86.2%) | <0.001 |
| Renal Disease, n (%) | 678 (17.5%) | 192 (20.9%) | 181 (19.5%) | 145 (15.4%) | 160 (16.8%) | 0.025 |
| Liver Disease, n (%) | 271 (7.0%) | 97 (10.5%) | 50 (5.4%) | 48 (5.1%) | 76 (8.0%) | <0.001 |

Data are presented as medians (interquartile ranges) for continuous variables or numbers (%) for categorical variables. Differences across quartiles were assessed via the Kruskal–Wallis test or chi-square test, as appropriate. Abbreviations are listed in Table 1.

**Table S4. Subgroup analyses for the associations between glycemic indices and incident NOAF within 7 days after ICU admission**

| **Outcomes exposure** | **Model 1** | | **Model 2** | | **Model 3** | |
| --- | --- | --- | --- | --- | --- | --- |
|  | **HR (95% CI)** | ***P*** | **HR (95% CI)** | ***P*** | **HR (95% CI)** | ***P*** |
| **DM** |  |  |  |  |  |  |
| **HGI group** |  |  |  |  |  |  |
| Q1 | Ref |  | Ref |  | Ref |  |
| Q2 | 1.01 (0.74-1.38) | 0.962 | 0.99 (0.72-1.35) | 0.951 | 1.05 (0.76-1.44) | 0.772 |
| Q3 | 0.77 (0.55-1.08) | 0.128 | 0.81 (0.58-1.13) | 0.223 | 0.90 (0.64-1.27) | 0.54 |
| Q4 | **0.39 (0.26-0.58)** | **<0.001** | **0.62 (0.41-0.94)** | **0.025** | 0.69 (0.44-1.06) | 0.091 |
| **SHR group** |  |  |  |  |  |  |
| Q1 | Ref |  | Ref |  | Ref |  |
| Q2 | 1.04 (0.74-1.46) | 0.825 | 0.83 (0.59-1.17) | 0.288 | 0.77 (0.54-1.10) | 0.153 |
| Q3 | 1.06 (0.76-1.50) | 0.727 | 0.84 (0.60-1.19) | 0.323 | 0.82 (0.58-1.17) | 0.278 |
| Q4 | 0.81 (0.56-1.17) | 0.258 | 0.70 (0.49-1.01) | 0.059 | 0.64 (0.43-0.94) | 0.024 |
| **No-DM** |  |  |  |  |  |  |
| **HGI group** |  |  |  |  |  |  |
| Q1 | Ref |  | Ref |  | Ref |  |
| Q2 | 1.25 (0.95-1.65) | 0.118 | 1.13 (0.85-1.49) | 0.398 | 1.13 (0.85-1.50) | 0.412 |
| Q3 | **1.74 (1.33-2.26)** | **<0.001** | **1.48 (1.14-1.93)** | **0.004** | **1.37 (1.04-1.81)** | **0.024** |
| Q4 | **1.83 (1.41-2.38)** | **<0.001** | **1.57 (1.20-2.04)** | **<0.001** | **1.37 (1.04-1.81)** | **0.024** |
| **SHR group** |  |  |  |  |  |  |
| Q1 | Ref |  | Ref |  | Ref |  |
| Q2 | **0.71 (0.56-0.90)** | **0.004** | **0.74 (0.58-0.93)** | **0.01** | 0.85 (0.67-1.08) | 0.181 |
| Q3 | **0.61 (0.47-0.77)** | **<0.001** | **0.66 (0.51-0.84)** | **<0.001** | **0.75 (0.58-0.96)** | **0.024** |
| Q4 | **0.60 (0.47-0.77)** | **<0.001** | **0.66 (0.52-0.84)** | **<0.001** | **0.67 (0.51-0.87)** | **0.003** |

Data are presented as hazard ratios (HRs) with 95% confidence intervals (CIs).
Model 1: unadjusted.
Model 2: adjusted for age, sex, BMI, and race.
Model 3: additionally adjusted for SOFA score, SAPS II score, ventilator use, CRRT, vasopressor use, vital signs (HR, MBP, RR, SpO2), laboratory parameters (WBC, RBC, platelet, chloride, calcium, potassium, sodium, creatinine), comorbidities (MI, CHF, DM, AKI, delirium, renal disease, liver disease), and cardiac surgery.

**Figure S1. Associations of the hemoglobin glycation index (HGI) and stress hyperglycemia ratio (SHR) with new-onset atrial fibrillation (NOAF), stratified by diabetes status (DM vs. non-DM), across three Cox regression models.**

Hazard ratios (HRs) are shown for quartiles of each glycemic index, with the lowest quartile (Q1) as a reference: (A) patients with diabetes (DM) and (B) patients without diabetes (non-DM). Within each panel, the results are presented separately for HGI (top) and SHR (bottom) under Models 1–3 (definitions and covariate adjustments provided in the Methods).


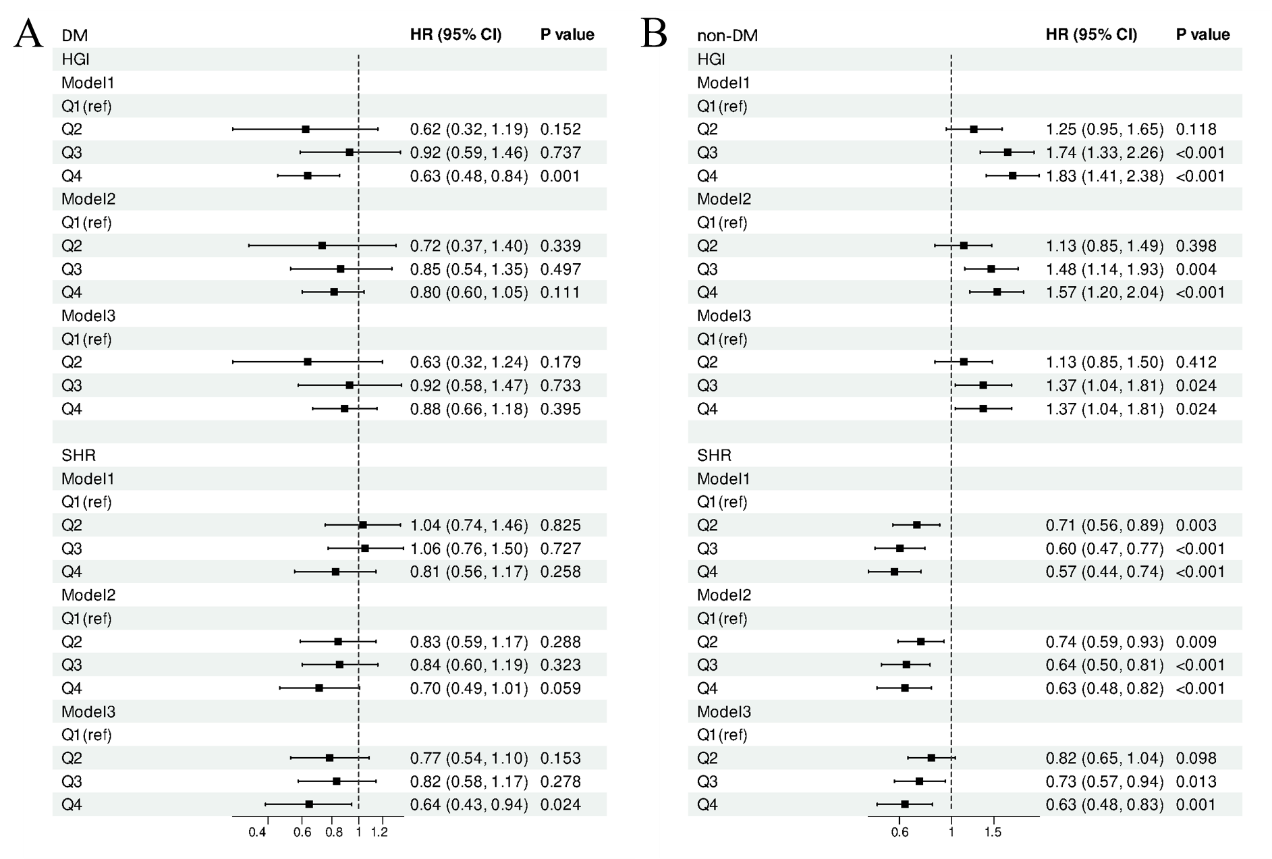

Supplement: Supplementary Table 1 — Additional baseline characteristics of critically ill patients with and without new-onset atrial fibrillation (NOAF). [file DataSheet1.docx]
